# Direct-to-consumer genetic testing

**DOI:** 10.1136/bmj.l5688

**Published:** 2019-10-16

**Authors:** Rachel Horton, Gillian Crawford, Lindsey Freeman, Angela Fenwick, Caroline F Wright, Anneke Lucassen

**Affiliations:** 1 Clinical Ethics and Law at Southampton (CELS), Faculty of Medicine, University of Southampton, UK,; 2 Wessex Clinical Genetics Service, Princess Anne Hospital, Southampton, UK; 3 Wessex Regional Genetics Laboratory, Salisbury, UK; 4 University of Exeter College of Medicine and Health, Exeter, UK

**What you need to know**

- Finding a “health risk” via direct-to-consumer (DTC) genetic testing often does not mean that a patient will go on to develop the health problem in question
- DTC genetic tests might report false positives (artefacts)
- “Reassuring” results from DTC genetic tests might be false negatives
- Make sure you are confident in the provenance and interpretation of a genetic result before you base any clinical decisions on it
- If your patient meets criteria for referral to clinical genetics, refer regardless of the results of their DTC genetic test

Direct-to-consumer (DTC) genetic tests are sold online and in shops as a way to “find out what your DNA says.”^1^ ^2^ Testing kits typically contain instructions and equipment for collecting a saliva sample, which customers post to the DTC company for analysis.

Some DTC genetic tests promise insights into ancestry or disease risks; others claim to provide information on personality, athletic ability, and child talent. However, interpretation of genetic data is complex and context dependent, and DTC genetic tests may produce false positive and false negative results.

Anyone concerned about a result from a DTC genetic test might turn to their general practitioner (GP) or other primary healthcare provider for advice. This practice pointer aims to help clinicians in this scenario and explains what sort of health information is provided by these tests, their limitations, and how clinicians can respond to common questions about them.

## What is a DTC genetic test?

Most DTC genetic tests don’t sequence the whole genome. They typically use a method called SNP-chip genotyping, which checks for the presence or absence of specific variants throughout the genetic code, such as particular single nucleotide polymorphisms (SNPs), or small insertions or deletions. SNP-chip genotyping detects common genetic variants well, but when SNP-chips detect very rare variants these are often false positives (ie, they are not really present in the person’s DNA).^3^

Genome sequencing is another method becoming more widely used in DTC genetic tests. These tests sequence almost the entire genetic code and identify the variants present within it. However, detecting variants is not the same as knowing their clinical effects—clinical interpretation of genetic variants is challenging and depends on context.

## The appeal of DTC genetic testing

People might be drawn to DTC genetic testing in the hope that it will provide clear cut information about their future health. This idea may be reinforced by advertising. For example, a recent analysis of advertising of DTC genetic tests noted that some tests were presented as potentially empowering, with the decision to take them portrayed as responsible—a way that people can take an active role in managing their own health.^4^ The “personalised medicine” that genetic testing promises is often portrayed in an optimistic light by the mainstream media,^5^ and genetic technology is generally presented as highly accurate. As a result, people may perceive genetic testing as clearly predictive, and expect that results will help them plan for the future.^6^ Our research group recently conducted a YouGov survey of around 2000 people in the UK, and found that the most common word that people chose to describe genome sequencing in healthcare was “informative.”^7^ A discussion with a patient considering having a genetic test is outlined in box 1.

Box 1 **Things you might discuss with a patient who is considering a DTC genetic test**

**Why do you want the test?**

If you have a specific clinical question (grounded in a personal or family history of a likely genetic condition) for which NHS genetic testing might be available, you would probably be better off accessing genetic testing via the NHS than via a DTC company. If you do not have a specific question, then discussing the pros and cons of particular tests is outside the scope of standard clinical practice.

**Imagine receiving a result you are concerned about**

Does the DTC genetic testing company have real people you can talk to? Are they qualified (eg, genetic counsellors) to advise in response to your clinical concerns?

If you are worried by the results of your test or want further advice, it might be hard to access this via the NHS.

**Have you read all the information and small print about the test?**

Sometimes tests have substantial limitations. If you are worried about a genetic condition in your family, is the DTC genetic test you are thinking about thorough enough to properly check this? (see *‘* *What are the limitations of DTC genetic tests?*)

**Could your decision to have a test affect your family?**

DTC genetic tests sometimes reveal information that could be relevant to your family—such as a health risk that might run in the family, or that family relationships are different from what you expected. Have you told your family that you are thinking about having a genetic test?

**Are you happy with what the DTC company might do with your data?**

DTC companies might collect, store, sell, or undertake research on your genetic data. Do you find that acceptable? Do you know who might have access to your data?

More information for people considering buying DTC genetic tests can be found on the Genetic Alliance website (https://www.geneticalliance.org.uk/information/service-and-testing/direct-to-consumer-genetic-testing/) and the Association of Genetic Nurses and Counsellors website (https://www.agnc.org.uk/info-education/documents-websites/).

## What health information do DTC genetic tests provide?

DTC genetic tests might provide a range of health information:

• **Polygenic risk scores**—combine many different common variants across the genome to place someone in a broad risk category, eg, “your genes predispose you to weigh about 3% more than average.” The validity and utility of these risk scores for predictive clinical purposes is hotly debated. In our opinion, although polygenic scores may be useful in researching the causes of disease, or stratifying populations into higher and lower risks, they are rarely able to usefully predict disease.^8^

• **Genotype at specific points**—looks at specific variants that influence the chance of developing particular diseases, eg, “you have two copies of the ε4 variant in the *APOE* gene. People with this result have an increased risk of developing late onset Alzheimer’s disease.” This type of testing can also be used to identify variants that affect drug metabolism.

• **Carrier screening**—looks at specific variants to identify people who are carriers for particular recessive genetic conditions, eg, “one variant detected in the *CFTR* gene. If you and your partner are both carriers, each child may have a 25% chance of having this condition.” Many carrier tests are ancestry specific: they test for specific carrier variants common in a particular ancestral group. If someone with a different ancestry were a carrier, this would probably not be detected as it would likely be due to a different carrier variant (which the test would not check).

• **Uninterpreted “raw” genetic data**—some DTC genetic test companies provide access to uninterpreted genetic data. Customers can download their data and seek an interpretation using third party services.^9^ These usually work by cross referencing the data against freely available genetic databases and constructing a report based on interpretations in these databases (which may not be up to date).^10^ They may report variants and disease risks that were not reported or referred to by the original DTC genetic test company, and might repurpose raw data from tests designed to answer other questions, such as ancestry, to try to provide health information.

## What are the limitations of DTC genetic tests?

### Predictive value is low when there is no family history of disease

*Jake bought a DTC genetic test online while researching his family history. He enjoyed learning about his ancestry so decided to pay for an optional health report. He was very upset to find that: “you have one variant detected in the LRRK2 gene. People with this variant have an increased risk of developing Parkinson’s disease.” Jake had no family history of Parkinson’s disease.*

If a “disease-causing” or “disease-predisposing” genetic variant is found in a person with no medical or family history of the corresponding disease, it may be that there are currently unmeasurable protective genetic (or other) factors in that person’s family that mean that the variant is less likely to lead to disease in that person. Most people with apparent “positive” results will not go on to develop the related condition.

The predictive meaning of a “disease-causing variant” is often much reduced when found outside the context of a family history of the relevant disease.^11^ For example, a study of people with a genetic form of diabetes found that up to 75% of those who carry a particular variant (R114W) in the *HNF4A* gene developed diabetes by age 40.^12^ A recent study looking at the same R114W variant in UK Biobank participants who were not pre-selected as having diabetes showed that only 10% developed diabetes by age 40.^11^ Even if a person does have a family history, identifying a “high genetic risk” via DTC genetic testing does not mean that they will definitely develop the condition.

### False positives are common, especially where third party interpretation services are used

*Aoife was given a DTC ancestry test for Christmas. She sent her raw genetic data to an online interpretation service. This reported that she had a disease-causing BRCA1 variant (increasing her risk of breast and ovarian cancer). Aoife did not have a strong family history of breast or ovarian cancer. She asked her GP to refer her to a breast surgeon. She met the surgeon, who booked a date for her surgery. Her GP also referred her to a clinical genetics service, which arranged NHS testing to check that the BRCA1 variant was really present in Aoife’s DNA—it wasn’t. The online interpretation service had reported a false positive result. Aoife’s operation was cancelled but she still felt anxious about her cancer risk.*

False positive results can arise for a number of reasons. The quality control for DTC genetic tests is variable. Some tests may be more vulnerable than NHS testing to issues like sample “miscalls,” where initial analysis appears to detect a particular genetic variant but subsequent scrutiny shows that it is an artefact. Results found via third party interpretation services need particular care. This is because the “raw data” that such services interpret will contain artefacts,^13^ and because the databases used to interpret the data may not be up to date (so might classify variants incorrectly based on outdated evidence).^10^

The SNP-chip genotyping method that most DTC genetic tests use is unreliable at testing for very rare disease-causing genetic variants. A recent study looking at *BRCA1* and *BRCA2* genes in UK Biobank participants found that 96% of disease-causing very rare variants identified by SNP-chip genotyping were false positives.^3^

### Reassuring results can be false negatives

*Lily had treatment for breast cancer in her 40s, and her mother died of ovarian cancer in her 50s. Lily was concerned about an inherited cause, but also wanted to help conserve NHS resources, so she bought a DTC test that included a BRCA screen. She was delighted by the result: no variants were found in BRCA1 or BRCA2. Lily’s GP explained that many DTC BRCA screens only screen for a small proportion of the possible variants in these genes, and referred her to clinical genetics in view of her family history. NHS genetic testing found that Lily had a BRCA1 variant that conferred a high lifetime risk of cancer.*

DTC genetic tests tend to prioritise breadth over detail. For example, the 23andMe “genetic health risk” report for *BRCA1* and *BRCA2* currently only checks for three disease-causing variants mainly relevant for people with Ashkenazi Jewish ancestry; this approach would miss in the region of 80% of people with disease-causing *BRCA* variants in the general population as there are thousands of different disease-causing *BRCA* variants that the test does not check for.^14^ ^15^ ^16^ ^17^

Widespread use of phrases such as “having the gene for X” means that many people think that there is just one *BRCA* test that checks if you’ve “got the *BRCA* gene.” This is incorrect: a *BRCA* test can range from a spot-check for a handful of specified variants, to a thorough examination of the sequence of both *BRCA* genes looking for a whole range of possible variants. If your patient has a personal or family history such that you would usually refer to clinical genetics, do this even if they have a reassuring result from a DTC genetic test.

## How do people interpret their DTC genetic test results?

Genetic data are complicated, and can easily be misinterpreted. DTC genetic tests are sold as providing answers, and patients may understandably expect that their results will be clearly predictive of future health. These expectations, driven by marketing and media coverage, leave people at risk of over-interpreting results from DTC genetic testing.

**Fig 1:**
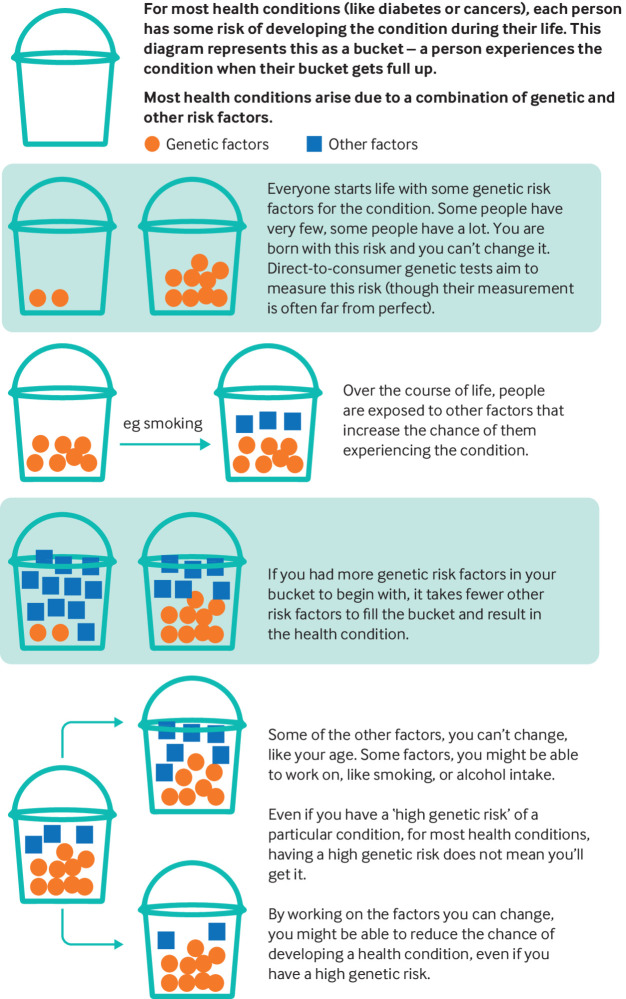
“Genetic risk” for common health conditions (adapted from Jehannine Austin’s “jar model” used in psychiatric genetic counselling^20^)

One common pitfall is to compare the result to a “zero risk,” rather than population risk. For example, the Secretary of State for Health declared that having a genetic test “may have saved my life,” after his polygenic risk score identified a 15% risk of developing prostate cancer by age 75. Experts disputed the usefulness of this result—and his interpretation of it—given that the average lifetime risk of a man developing prostate cancer is 18%, and more men die with, than from, prostate cancer.^18^ Careful framing of results (for example, comparison with population risks) may mitigate the risk of over-interpretation. However, this relies on information being provided in an accessible manner (fig 1); furthermore, users need to know how important it is to read the information, which may not be obvious in the context of a societal discourse, which tends to present genetic results as strongly predictive. The assumption that DTC genetic testing empowers people to reduce their future disease risk is undermined by evidence that suggests that learning about genetic predisposition to particular diseases rarely leads to sustained lifestyle change.^19^

DTC genetic tests are often accessed without discussion with a health professional, so there is little opportunity to address potential mismatches between expectation and reality in advance of testing. Often, relevant and important information regarding the limitations of tests is available on DTC genetic testing websites, but it is easy to see how their disclaimers might be perceived as extensive and tedious lists of terms and conditions, which many people might not engage with in detail.

## When should you consider onward referral?

Patients might ask you about a wide range of “positive” results—most will not need referral to clinical genetics. Your regional clinical genetics service may have a local policy regarding DTC genetic test results, and guidance from the Royal College of General Practitioners is expected to be published later this year.

DTC genetic test results should not be used to inform health decisions without further scrutiny. For patients said to have “disease-causing” or “likely disease-causing” variants in genes associated with conditions for which early detection may be possible and/or treatment is available (eg, *BRCA1*, *BRCA2*, *MLH1*), inform the patient that further testing often shows that DTC genetic test results are inaccurate, so they might need a blood test to confirm or refute their result, and they might not be offered a genetics appointment.

Refer anyone with a medical or family history where you would otherwise offer a genetics referral (ie, refer regardless of “reassuring” results from a DTC genetic test). If in doubt, contact your regional clinical genetics service to discuss whether referral is appropriate.

If a patient who has taken a DTC genetic test presents with symptoms that they are concerned about (or that they think are explained by the DTC genetic result), assess and investigate the symptoms in the same way that you would for any other patient. If the DTC genetic result would change a patient’s clinical management if it were correct, it needs to be confirmed in an accredited laboratory.

## What to say to patients

**Fig 2:**
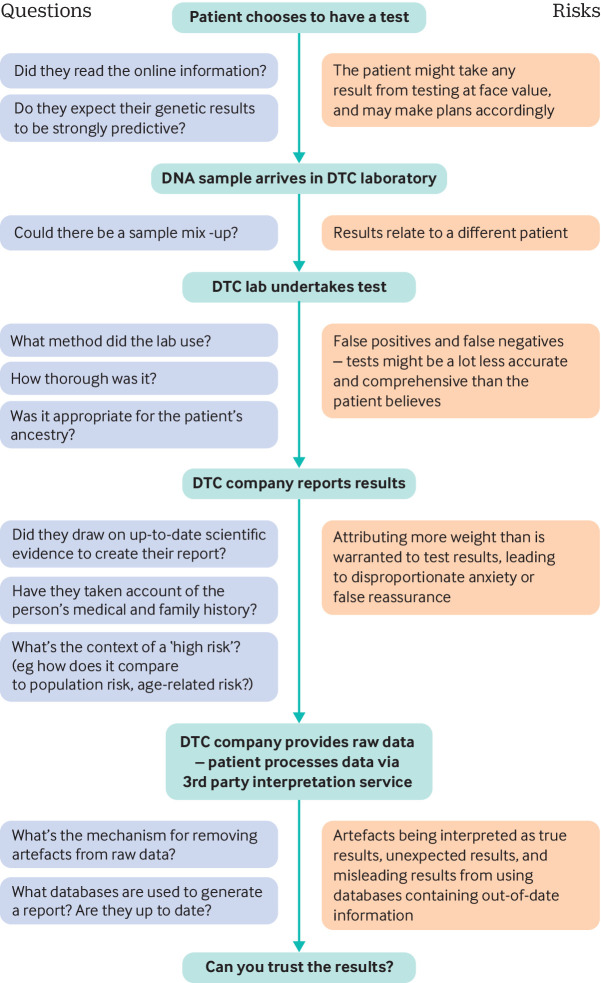
The process of DTC genetic testing, from consumer decision to test through to receiving results

When a patient presents with a DTC genetic test result, they may find an explanation of some of the issues discussed in this article helpful. Patients might understandably take results at face value, so it may be useful to discuss possible sources of error in DTC genetic tests (fig 2). Shifting the conversation from discussing genetic risk to addressing modifiable lifestyle factors can be an important part of the consultation. 

**How patients were involved in the creation of this article**

We met the Patient and Public Involvement group at University Hospitals Southampton NHS Foundation Trust to discuss how patients might react to receiving concerning DTC test results, their expectations as to how the NHS should respond, and their views regarding what information might be helpful to discuss with patients. These discussions helped inform every section of the article but were especially key for shaping the section “What to say to patients.” We had 38 responses to a survey on social media about what people might expect from DTC genetic tests, and how they might respond to results. This helped inform the sections “What might people expect from DTC genetic tests?” and “Wider questions,” which reflect some of the issues raised by the survey respondents.

**Education into practice**

- What might you ask if a patient told you they were considering buying a DTC genetic test?
- What might you say to a patient who booked an appointment to discuss a DTC genetic test result indicating a high genetic risk of prostate cancer?
- What would you say to a patient who started smoking again after receiving a reassuring health report from a DTC genetic test?

**Wider questions**

- How can we raise public awareness that many genetic results are not clear cut, and may not substantially shift a person’s pre-existing risk of disease?
- How can we ensure that patients with personal or family histories suggesting a genetic condition are not falsely reassured by DTC genetic test results?
- How should additional healthcare costs arising from DTC genetic tests be funded? What regulation might be helpful?

**How this article was made**

We developed fictitious cases to illustrate some of the issues with DTC genetic tests, based on recent referrals to our regional genetics department, discussions at GenethicsUK (a forum to discuss ethical issues in genomic medicine – www.genethicsUK.org), and cases notified to the British Society of Genomic Medicine. Two of the vignettes discuss *BRCA* results as referrals to clinical genetics about DTC genetic test results currently most commonly relate to *BRCA* variants.

Information relating to the results that DTC genetic tests commonly provide, and to the technology commonly used, is based on accessing websites for various popular DTC genetic testing companies. DTC genetic testing is a rapidly expanding market and many different tests are available, which use various analyses and reporting practices, so inevitably the information in this article cannot be comprehensive.

RH drafted the article; GC edited clinical cases, referral criteria, and facilitated PPI involvement; LF contributed to clinical cases and fig 2; AF edited the article and advised on development of figures; CW adapted the article to improve technical accuracy; AL conceived of article following cases arising in clinical practice and edited draft as a whole.

## Notes

**Acknowledgments** Thanks to the National Institute for Health Research (NIHR) supported Patient and Public Involvement group at the NIHR Clinical Research Facility and NIHR Biomedical Research Centre at University Hospitals Southampton NHS Foundation Trust, all the people who responded to our Twitter survey, Caroline Barker and Michael Bahrami-Hessari (NIHR CRF), and Charlotte Forder (Wessex Academic Health Sciences Network) for their input on this article.

**Patient consent** The case(s) in this article are fictitious and therefore no consent was needed.

Provenance and peer review: commissioned; based on an idea from the authors.
